# Outcomes from Individual Alpha Frequency Guided Repetitive Transcranial Magnetic Stimulation in Children with Autism Spectrum Disorder – A Retrospective Chart Review

**DOI:** 10.1007/s10578-022-01461-1

**Published:** 2022-11-11

**Authors:** Uchenna Ezedinma, Piotr Swierkowski, Shauna Fjaagesund

**Affiliations:** 1 Brain Treatment Centre, 19-31 Dickson Road, Morayfield, QLD Australia; 2 University of the Sunshine Coast, Sippy Downs, Australia

**Keywords:** Autism spectrum disorder, Children, Electroencephalogram/electrocardiogram, Repetitive transcranial magnetic stimulation, Neuromodulation

## Abstract

**Aims and objectives**: Individual alpha frequency (IAF) is a biomarker of neurophysiological functioning. The IAF-guided repetitive transcranial magnetic stimulation (α-rTMS) is increasingly explored in diverse neurological conditions. However, there is limited data on the efficacy and safety of α-rTMS in children with autism spectrum disorder (ASD). **Materials and methods**: The IAF, childhood autism rating scale (CARS), Pediatric Quality of Life Inventory 4.0 (PedsQLTM 4.0), and semi-structured interview data of patients that received 19 α-rTMS sessions (4 weeks) were aggregated and analysed using paired student t-test and descriptive method. **Results**: Data were retrieved from 28 patients (26 males, aged 3-9years (mean ± SD age: 6.1 ± 1.8years)). The post-α-rTMS data shows a significant improvement in IAF (9.4 Hz; *p* *≤* *0.025*) towards 10 Hz. The CARS and PedsQLTM 4.0 surveys indicate that patients’ ASD symptoms and quality of life improved significantly. Specifically, reports from semi-structured interviews suggest improved sleep trouble – the most significant comorbidity. The experiences of minor side effects such as hyperactivity resolved within two hours following α-rTMS sessions. **Conclusion**: This study presents evidence on the efficacy and safety of α-rTMS in improving ASD symptoms, quality of life and comorbid sleep troubles in children. However, these findings should be interpreted as preliminary pending the presentation of double-blind, randomised clinical trials.

## Introduction

Autism spectrum disorder (ASD) is an increasingly prevalent neurodevelopmental disorder that impacts about 1% of children worldwide [1]. In Australia, 7.1% of children (0–14 years old) in 2018 were diagnosed with ASD, compared to 6.0% in 2015, with a prevalence four times higher in males than females [2]. Diagnosis is based on marked deficits in social and communication skills and restricted and repetitive behaviour patterns [1, 3, 4]. These symptoms may present with and worsen by comorbidities such as seizures, sleep troubles, anxiety, and attention-deficit/hyperactivity disorders (ADHD) in children [2, 3, 5–7]. Such constellations of ASD symptoms and comorbidities lend to different levels of ASD diagnosis and determine the quality of life [8, 9].

Several early evidence-based interventions designed to improve ASD symptoms and comorbidities have been limited, inconclusive, and associated with nocebo effects [1, 3, 10]. There are renewed interests in applying non-invasive brain stimulation such as repetitive transcranial magnetic stimulation (rTMS) in alleviating symptoms and comorbidities associated with ASD due to its modulating effect on cortical plasticity and inhibition, aberrant cortices, and a lasting effect of six months [11–15]. However, the heterogeneous presentation of ASD, such as variabilities in age, ASD symptom severity, and comorbidities, amongst others, limits the reliable measure of rTMS efficacy and safety and translation into clinical practice [11, 12].

Consequently, there is a growing consensus on the need to individualise rTMS protocols toward delineating the heterogeneous nature of ASD presentations [12]. Recent studies are investigating electroencephalogram/electrocardiogram (EEG/ECG) use in individualising rTMS protocols due to its correlation with patient’s neurophysiological functioning [11, 16–20]. For instance, EEG/ECG studies show that individual alpha frequency (IAF) within the frontal, central, temporal and occipital regions of children with ASD are delayed or incoherent compared to typically developing children of similar age [14, 21, 22]. An IAF-guided rTMS (α-rTMS) is a promising modality, but there is limited evidence of its efficacy and safety in children with ASD [11, 23].

## Methods

Guided by Gearing’s nine-step for conducting retrospective chart review research, we reviewed the clinic data of children with ASD who received 19 α-rTMS sessions between November 2018 and August 2022 at two Brain Treatment Centres in Queensland, Australia. [24]. Clinic data include patient’s IAF, Childhood Autism Rating scale (CARS) [25], Paediatric Quality of Life Inventory 4.0 (PedsQL 4.0) [8] and semi-structured interviews before and after 19 α-rTMS sessions (four weeks) [26]. The CARS and PedsQLTM 4.0 were self-administered by the patient’s primary caregivers to minimise potential response bias [27], while observations of the patient’s ASD symptoms, comorbidities, and any side effects were obtained from primary caregivers at semi-structured interviews before and after α-rTMS. Given the advantages of α-rTMS, we include data from patients of all ages, sex, ASD categories and comorbidity but excluded co-diagnoses of neurocognitive and or congenital conditions such as intellectual disorder, Down syndrome, Fragile X, Klinefelter syndrome, angleman syndrome and Prada Willis syndrome [12], The primary caregivers provided informed written consent and held knowledge that α-rTMS use in ASD is *off-label.*

Following EEG/ECG conducted on the TruScan acquisition software (Deymed diagnostic, s.r.o, Czech Republic), the patient’s IAF (stimulation frequency) and stimulation sites were determined using the methodology described by Taghva et al. [26]. Briefly, EEG/ECG time series were converted to frequency-domain using Fast Fourier Transform (FFT). The stimulating frequency was determined by identifying the dominant peak frequency with the highest power in the 8-13 Hz range and multiplying it by the higher harmonic frequency (5th to 10th ) of the ECG nearest to the dominant peak frequency. The stimulation sites were determined by identifying the brain region with the highest aberrant cortical processes compared to a normative database with equal parameters and measured using the 10–20 system [23, 26]. The α-rTMS was a 5-second stimulation train with pulses delivered at calculated IAF with 28-second intervals between 32 trains per cortical site using an MCF-B65 butterfly coil and Magpro R30 TMS stimulator (Magventure Inc, Denmark) [23, 26]. The resting motor threshold was determined by placing the centre of the coil on the motor cortex of the patient and gradually increasing the output of the TMS machine by 5% until a visible twitch in the muscle of the contralateral fingers was observed in two out of three trials [28]. The output intensity of α-rTMS was administered at 80% of the resting motor threshold to minimise potential side effects [26, 29]. Each α-rTMS session was administered during the weekdays and lasted approximately 40 min a day, with patients allowed to colour-in artworks or snack between stimulation trains to enable compliance.

This study received ethics approval from the University of Southern Queensland, Australia, with registration number H21REA177.

### Statistical Analysis

The patient’s IAF, CARS and PedsQLTM 4.0 data were aggregated and analysed using a paired student t-test with confidence intervals of 95% and Cohen’s D effect size (ES) for statistical significance. A descriptive presentation of data from semi-structured interviews was conducted. With all patients receiving equal α-rTMS sessions and at different times, any consistent and significant changes following statistical analysis are due to α-rTMS rather than by chance [24, 30].

## Results

The clinic data of 28 patients (26 males) aged 3-9years (mean 6.1 ± 1.8) were retrieved. Ten and eighteen patients were diagnosed with ASD levels 2 and 3, respectively, between the ages of 1.5-7yrs (mean 3.2 ± 1.5). Frequently reported comorbidities by patient’s primary caregivers includes sleep troubles (n = 17), anxiety (n = 3), ADHD (n = 2) and seizure (n = 1). Patient’s medical history shows the use of medications such as melatonin (n = 6), multivitamins and supplements such as zinc, vitamin B and D, iron, magnesium (n = 7), methylphenidate (n = 3), risperidone (n = 1), naturopath/traditional medicine (n = 2), Nemechek protocol (n = 1), clonidine (n = 1), and cannabidiol (n = 1) while alternative intervention that were accessed includes speech (n = 10), occupational (n = 12), cognitive behaviour (n = 1), and sound (n = 1) therapies, psychology (n = 3) services, and applied behavioural analysis (n = 1). Six patients did not report using or accessing medication and alternative pharmacological (Table 1).

**Fig. 1:**
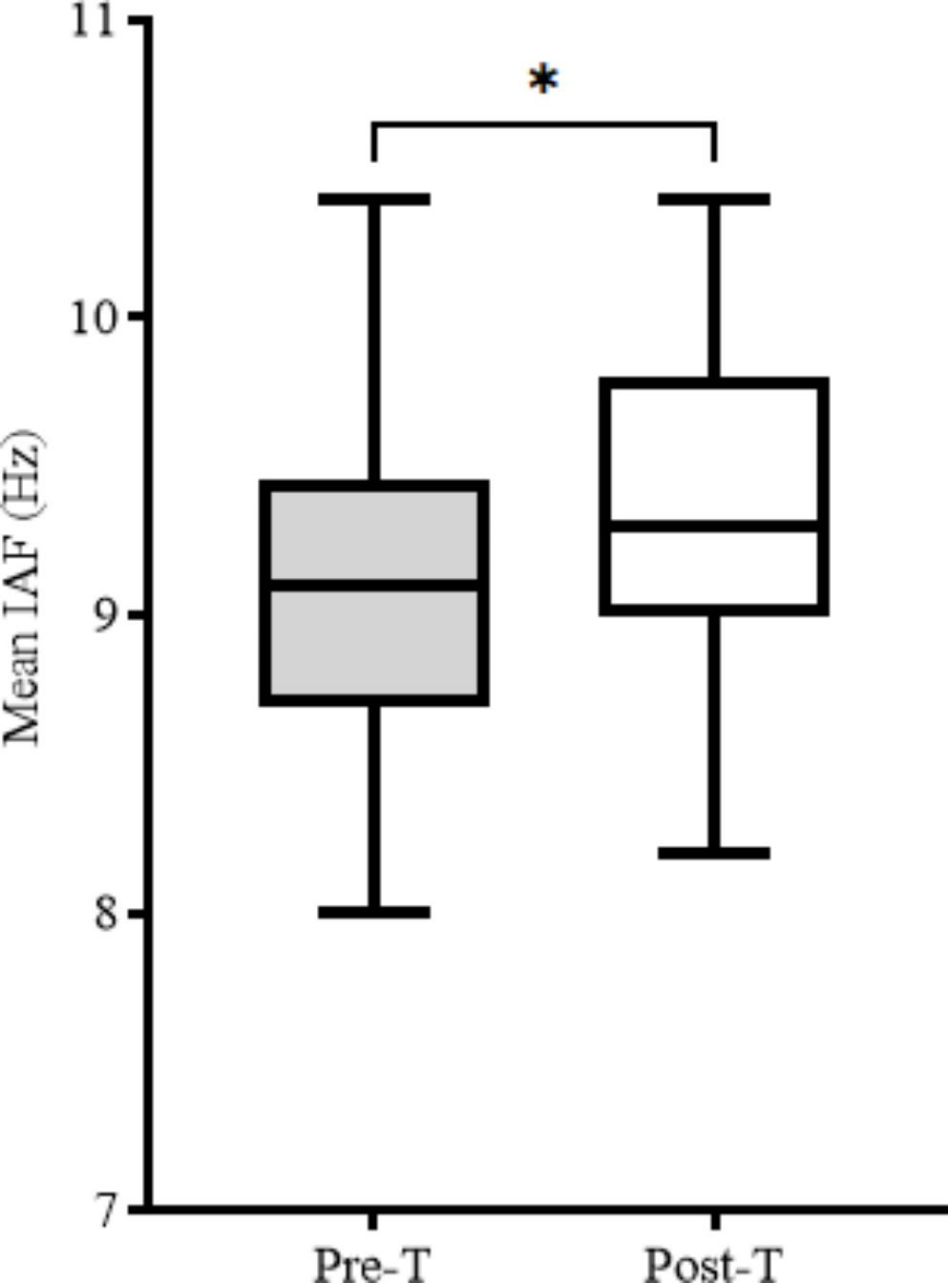
Mean IAF data. Pre-T: Pre-treatment, Post-T: Post-treatment, (*): p-value ≤ 0.05

**Fig. 2:**
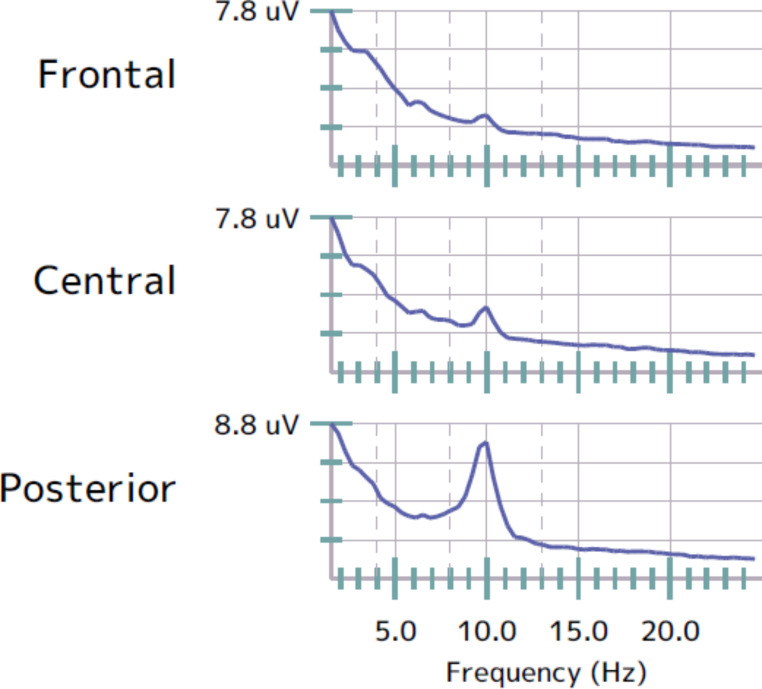
Sample representation of patient’s EEG/ECG. The aberrant cortical process (attenuated peak within the alpha band at the frontal region) was identified for FPz stimulation only

The pre- α-rTMS IAF was 9.1 Hz ± 0.6. Following 19 α-rTMS administered to the midline sagittal plane of the prefrontal lobe (FPz) and or midline parietal region (Pz) cortical sites (FPz/Pz (n = 27) and (FPz (n = 4)), there was a significant shift in the mean post- α-rTMS IAF to 9.4 Hz ± 0.6 with a *p-value of≤* *0.025* and small effect size of 0.4 (Table 1: Figures 1 and 2).

**Table 1:** Summary of patient’s treatment data

Patients	Sex	Age (yrs)	Diagnosis age (yrs)	ASDs level	Comorbidities	Medications	Alternative intervention	Pre-T (Hz)	Post-T (Hz)	Stimulation site	Reported outcomes
1	M	3	1.5	3	ST	-	ABA	9	9.2	Pz Fpz	Improved eye contact, independency, spatial awareness, sleep, vocalisation, physical activity, and mood
2	M	3	1.5	3	ST	Melatonin	SP, OT	9.5	9.8	Pz Fpz	Improved sleep, speech articulation and expression, imitation, physical activity, confidence, eye contact, spatial awareness, cognition, and non-verbal communication. A case of hypersalivation and “tongue-twisting”.
3	M	3	3	2	-	Iron and vitamin B	-	8.7	9.1	Pz Fpz	Improved anxiety, spatial awareness, listening skills, and vocalisation. Nil changes to eye contact.
4	M	3	2	3	ST	Melatonin	SP, OT and psychology	8.2	8.9	Pz Fpz	Improved vocalisation, non-verbal communication, spatial awareness, tactile skills, social skills, sleep, eye contact, anxiety, behaviour (aggression)
5	M	4	2	2	ST	-	-	8.7	8.4	Pz Fpz	Improved vocalisation, physical activity, eye contact, social skills, sleep, concentration, and anxiety. Nil changes in communication skills.
6	M	5	2	3	-	-	-	9.3	9.3	Pz Fpz	Improved cognition, social skills, vocalisation, tactile skills, eye contact, and anxiety. Nil changes to diet diversity and speech articulation.
7	F	5	3	3	-	-	-	9.3	9.3	Pz Fpz	Improved listening, handwriting, and spatial awareness. More tantrums and screaming (sensory overload).
8	M	5	3	3	ST	Melatonin	CBT and OT	10.1	10.2	Pz Fpz	Improved curiosity and concentration, spatial awareness, and social skills. Nil changes to sleep and speech.
9	M	5	3	3	-	Naturopath	SP, OT and psychology	9.3	9.3	Pz Fpz	Improved emotional regulation, eye contact, social skills, spatial awareness, and vocalisation.
10	M	5	3	3	ST	Vitamin B	Craniosacral stimulation	9.2	9.1	FPz	More emotional (crying for no reason)
11	M	6	4	3	Seizure	Cod liver oil, zinc, vitamin D, Iron	-	9.1	10.4	Pz Fpz	Improved anxiety, social skills, physical activity, vocalisation, imitation, behaviour, and concentration.
12	F	6	2.5	3	ST	-	-	8.8	9.1	Pz Fpz	Improved sleep, mood, vocalisation, and physical activity. Nil changes to the intensity of emotion and anger outbursts.
13	M	6	6	2	ST	Naturopath	-	8	10.4	Pz Fpz	Improved decision-making and mood. Increased screaming (due to more awareness or sensory overload)
14	M	6	3	2	ST, ADHD	Methylphenidate, risperidone, clonidine, melatonin	-	8.7	9.1	Pz Fpz	Improved anxiety, verbal communication, emotional regulation, phobia, and social skills
15	M	6	3.5	2	-	-	SP, OT	9.8	10.3	Pz Fpz	Improved speech, emotional regulation (anger), anxiety and behaviour.
16	M	6	3	3	ST	Melatonin	Nemechek protocol	9	8.8	Pz Fpz	Improved mood, concentration, verbal and non-verbal expression, sleep, and diet diversity. Nil changes to physical activity.
17	M	7	1	3	ST, anxiety	Melatonin, CBD, multivitamins	-	8.5	8.2	Pz Fpz	Improved social skills, cognition, self-expression, mood, and diet diversity.
18	M	7	4	3	ST, anxiety	-	SP, OT	10.4	9.8	Pz Fpz	Improved anxiety, repetitive behaviour, and listening skills.
19	M	7	6	2	ST	-	-	9.1	9	Pz Fpz	Improved sleep, diet diversity, anxiety, phobia. Nil changes to emotional dysregulation.
20	M	7	4	3	ST	Methylphenidate	-	8.7	9.5	Pz Fpz	Improved social skills, behaviours (aggression and meltdown), sleep and vocalisation.
21	M	7.5	2	3	-	Zinc, Magnesium	-	9.3	9.3	Fpz	Improved eye contact, vocalisation, mood, sleep, listening response, spatial awareness, and emotional regulation.
22	M	8	2.5	3	-	-	OT	10.3	10.3	Pz Fpz	Improved anxiety, phobia, concentration, listening response, and verbal communication.
23	M	8	4	2	-	-	SP, OT	9.1	9.6	Pz Fpz	Improved sleep, emotional regulation, and concentration.
24	M	8	7	1	ST, anxiety	-	SP, OT and psychology	8.7	8.7	Pz Fpz	Improved social skills, emotional dysregulation and behaviour (aggression).
25	M	9	2	3	ST	-	SP, OT and sound	9.2	8.8	Fpz	Improved spatial awareness, concentration, social skills, eye contact, listening response, physical activity, and behaviour.
26	M	9	5	3	ST	-	-	9.5	9.7	Pz Fpz	Improved cognition, eye contact, and special awareness. Nil changes in sleep and verbal communication.
27	M	8	4	3	ADHD	-	SP, OT	10	9.9	Pz Fpz	Improved mood, eye contact, verbal communication, and anxiety. Nil changes to physical activity.
28	M	9	1.5	2	-	Methylphenidate	SP, OT	9	9	Pz Fpz	Improved communication, mood, and behaviour. Nil changes to listening response.

**Key**: Not listed (-); *Applied behaviour analysis (ABA), Attention deficits hyperactive disorder (ADHD), Cannabidiol (CBD); Cognitive behavioural therapy (CBT), Occupational therapy (OT), posttraumatic stress disorder (PTSD), Sleep trouble (ST), and Speech therapy (SP): Pre-treatment (pre-T): Post-treatment (post-T)*

**Fig. 3:**
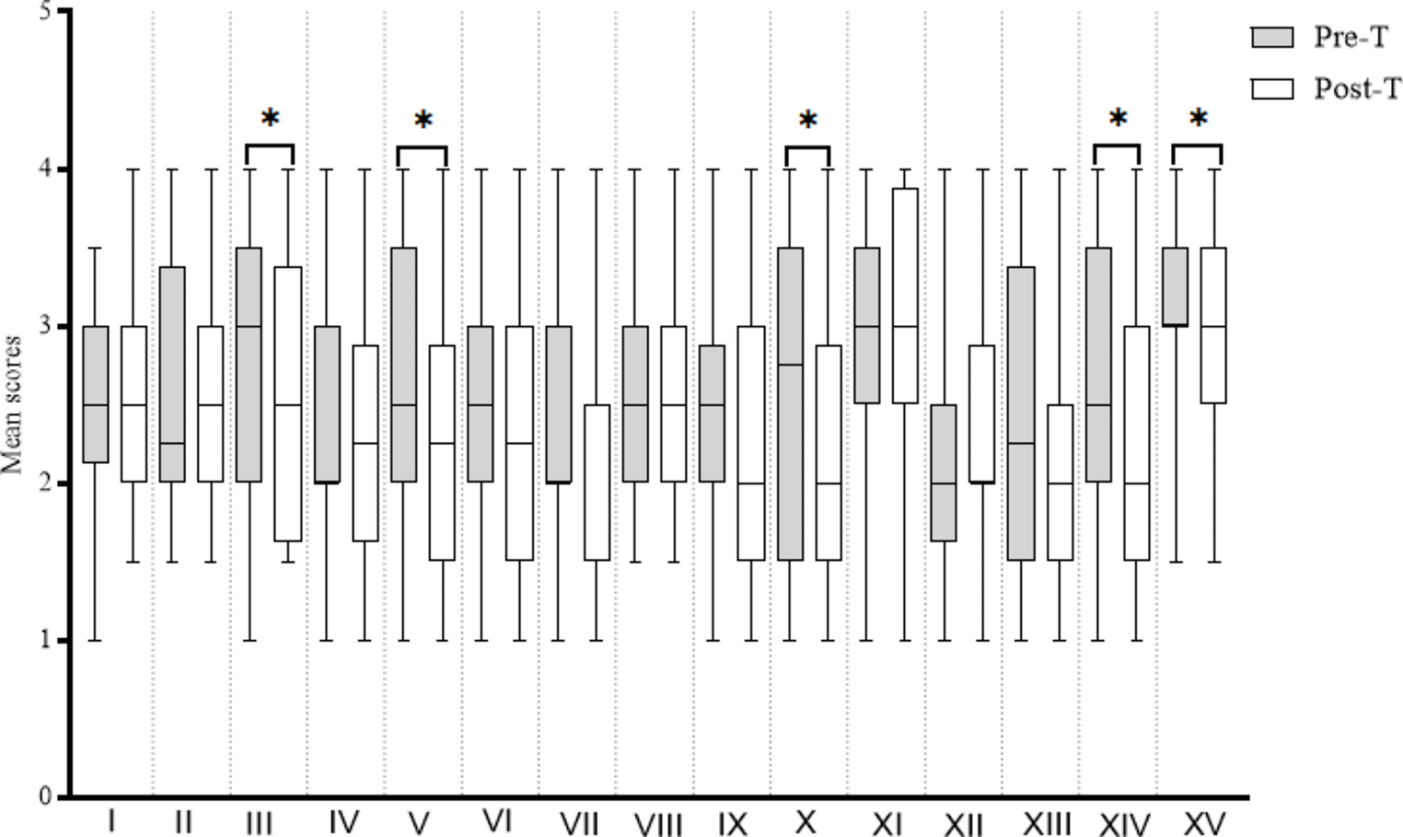
Mean score of CARS domains. Relating to people (I), Imitation (II), Emotional response (III), Body use (IV), Object use (V), Adaptation to change VI), Visual response (VII), Listening response (VIII), Taste/smell/touch response and use (IX), Fear or nervousness (X), Verbal communication (XI), Non-verbal communication (XII), Activity level (XIII), Level of consistency of intellectual response (XIV), General impression (XV), Pre-T: Pre-treatment, Post-T: Post-treatment, (*): p-value ≤ 0.05

Table 2 shows that the mean score of pre-treatment CARS was higher (> 2.5 points) in domains such as relating to people (2.61), imitation (2.52), emotional response (2.75), object use (2.54), listening (2.55), fear or nervousness (2.57), verbal communication (3.07), consistency of intellect (2.59) and general impression (3.14). Following treatment, the emotional response (p ≤ 0.05, ES:0.3), object use (p ≤ 0.05, ES:0.3), fear or nervousness (p ≤ 0.03, ES:0.3), level of consistency of intellectual response (p ≤ 0.02, ES:0.4) and general impression (p ≤ 0.04, ES:0.3) domains were statistically significant with a small to medium effect size (ES). The total CARS score at pre-and-post treatment was 38.2 + 0.3 and 36.6 + 0.3, respectively (Table 2: Fig. 3).

**Table 2:** Summary of CARS analysis

CARS domain	Pre-treatment Mean + SD	Post-treatment Mean + SD	*P value*	Effect size
Relating to people	2.6 + 0.6	2.5 + 0.5	*0.1*	0.1
Imitation	2.5 + 0.9	2.5 + 0.7	*0.5*	0
Emotional response	2.8 + 0.8	2.5 + 0.8	*0.05*	0.2
Body use	2.3 + 0.9	2.3 + 0.8	*0.5*	0
Object use	2.5 + 1	2.3 + 0.9	*0.05*	0.2
Adaptation to change	2.4 + 0.8	2.3 + 0.9	*0.2*	0.1
Visual response	2.3 + 0.9	2.3 + 0.7	*0.3*	0.06
Listening response	2.6 + 0.7	2.5 + 0.7	*0.2*	0.09
Taste, smell, and touch response and use	2.3 + 0.7	2.4 + 0.9	*0.4*	-0.02
Fear or nervousness	2.6 + 1.0	2.3 + 0.9	*0.03*	0.3
Verbal communication	3.07 + 0.8	3.1 + 0.8	*0.3*	-0.04
Non-verbal communication	2.2 + 0.8	2.3 + 0.7	*0.2*	-0.01
Activity level	2.3 + 1.0	2.1 + 0.9	*0.06*	0.2
Level and consistency of intellectual response	2.6 + 0.8	2.3 + 0.9	*0.02*	0.3
General impressions	3.1 + 0.6	2.9 + 0.7	*0.04*	0.2
Total CARS score	38.2 + 0.3	36.6 + 0.3		

**Fig. 4:**
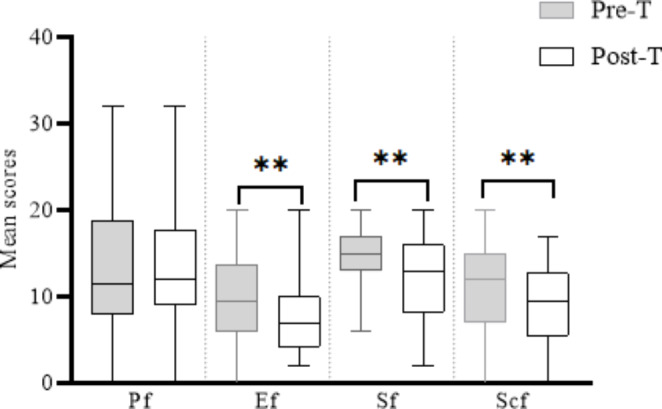
Mean score of PedsQLTM 4.0 subscales. PF: Physical function, EF: Emotional function, SF: Social function, ScF: School function, Pre-T: Pre-treatment, Post-T: Post-treatment, (*): p-value ≤ 0.05

Table 3 shows the mean score of the physical, emotional, social, and school functions of the pre-treatment PedsQLTM 4.0 survey to be 13.32, 9.54, 14.61, and 11.18, respectively. However, analysis of post-treatment data shows a statistical significance and a small to medium effect size (ES) within the emotional (p ≤ 0.007, ES:0.4), social (p ≤ 0.005, ES:0.6) and school (p ≤ 0.01, ES:0.4) functions. The total PedsQLTM 4.0 score at pre-and-post treatment was 48.6 and 41.4, respectively (Table 3: Fig. 4).

**Table 3:** Summary of PedsQLTM 4.0 analysis

PedsQLTM 4.0 subclass	Pre-treatment Mean + SD	Post-treatment Mean + SD	*P value*	Effect size
Physical functioning	13.3 + 8.2	12.8 + 7.8	*0.3*	0.06
Emotional functioning	9.5 + 5.4	7.5 + 4.8	*0.007*	0.4
Social functioning	14.6 + 3.4	11.9 + 4.9	*0.005*	0.6
School functioning	11.2 + 5.4	9.1 + 5.4	*0.01*	0.4
Total PedsQLTM 4.0 score	48.6 + 2.3	41.4 + 2.5		

A descriptive presentation of reported outcomes from semi-structured interviews with the patient’s primary caregiver identified the following common improvement such as social skills - eye contact; behavioural response – reduced aggression, anxiety, phobia and emotional dysregulation; verbal communications – increased vocalisation/bubbling/mumbling or more worded sentences; cognitive skills – increased spatial awareness, concentration, decision making and tactile skills; and general wellbeing - improved sleep, dietary diversity and physical activity. There was no report on the traditional side effects of α-rTMS, such as headaches and discomfort at the stimulation site. However, hyperactivity/tantrums/crying/screaming and a rare case of hypersalivation and tongue-twisting that resolved within 2 h following treatment were reported. Neither the primary caregivers nor the clinicians observed any seizures during treatment (Table 1).

## Discussions

High-frequency rTMS (> 5 Hz) are known to potentiate reduced levels of cortical plasticity and inhibition typical of children with ASD [11, 12, 31]. With α-rTMS administered at ≥ 9 Hz to all patients (mean age: 6yo), the significant shift in IAF towards 9.4 Hz indicates the potentiation of aberrant cortical processes towards frequency comparable to typically developing children of similar age [21, 22]. More so, such potentiation toward 10 Hz correlates with improvements in the patient’s neurophysiological functioning [11, 16–22]. For instance, evidence from the CARS survey shows a significant reduction in the degree of ASD symptoms from severe (> 36.5) to mild to moderate (30-36.5), while data from the PedsQLTM 4.0 suggest a significant improvement in the quality of life of the patients [8, 25].

The findings from the CARS and PedsQLTM 4.0 survey are corroborated by frequent reports from semi-structured interviews on improved eye contact, dietary diversity, environmental awareness, and comorbid sleep trouble [3, 5–7, 32–34]. Specifically, the report of improved sleep trouble is a novel and significant outcome, given the correlation between the prevalence of comorbid sleep trouble and the diagnosis of severe ASD (level 3) in most patients [3, 5, 9]. This finding also espouses the existence of a bi-directional outcome between ASD symptoms and comorbid sleep trouble following α-rTMS [15, 19]. Amongst the medication and alternative intervention-naïve patients (n = 6, 21%), and with the inefficacy of frequently used melatonin amongst medicated patients, α-rTMS may be a potential alternative first-line intervention for comorbid sleep trouble in ASD [1, 3, 10, 35].

The identified aberrant cortical sites (FPz/Pz and FPz) parallel findings from previous studies [11, 12]. The value of EEG/ECG in identifying aberrant cortices is a significant factor in individualising treatment protocol and ensuring the α-rTMS effect across patients with heterogeneous characteristics [12, 20, 26]. Given that the FPz and Pz sites underpin emotional inhibition, modulation of emotional (sensitivity) and behavioural responses, motivation /attention and working memory, and the integration of somatosensory information with posterior visual perceptions, respectively, potentiating these cortices may support improved ASD symptom as evident on the post- α-rTMS IAF data [12, 14, 23]. However, based on the principles of cortical plasticity and shared neuropathologies, other distal or subcortical sites that mediate neurophysiological functions such as sleep may be modulated and thus, underscore the α-rTMS effect on sleep trouble [15, 19, 29, 31, 36].

The α-rTMS effects may be supported by the advantages of early ASD diagnosis (mean age 3 years), access to α-rTMS (mean age 6 years) and the sex-influenced neural circuitries amongst males [2, 4, 37, 38]. With all patients receiving approximately 20 α-rTMS sessions, the improved ASD symptoms and comorbid sleep troubles may last longer than the six months identified in rTMS [13]. Despite the prolonged session and high-frequency stimulation of α-rTMS on potentially multiple brain regions, the absence of adverse effects suggests its safety in children (3-9years) with ASD, even with a history of seizure or concomitant medication use such as methylphenidate [6, 39]. However, due to communication deficits in most patients, traditional side effects such as headaches that resolved within two hours may have been reported as hyperactivity/tantrums/crying/screaming.

### Limitations

Due to the inherent limitations of retrospective studies [24], there is a need for future prospective studies to fully evaluate the effects and safety of α-rTMS in children with ASD. Specific limitations of this study include the small sample size, lack of objective measures for improved sleep troubles [15], the influence of concurrent interventions such as speech and occupational therapies, and methylphenidate and melatonin on patient’s IAF [40] and clinical outcomes [41–43] and a post-study follow. Future studies may also measure the quality of life of patients’ primary caregivers following α-rTMS [5].

## Summary

Autism spectrum disorder (ASD) is a neurodevelopmental disorder with significant comorbidities such as sleep troubles, especially in children. The use of repetitive transcranial magnetic stimulation (rTMS) as an alternative treatment for ASD in children is promising but limited by the heterogeneous nature of ASD presentation that undermines the efficacy of a standard treatment protocol. Data from electroencephalogram (EEG) studies such as individual alpha frequency (IAF) is increasingly explored as a biomarker for individualising rTMS treatments. The efficacy and safety of IAF-guided repetitive transcranial magnetic stimulation (α-rTMS) has not been demonstrated in children with autism spectrum disorder (ASD).

This study reviewed IAF, childhood autism rating scale (CARS), Pediatric Quality of Life Inventory 4.0 (PedsQLTM 4.0), and semi-structured interview data of patients that received 19 α-rTMS sessions (4 weeks) were aggregated and analysed using paired student t-test and descriptive method. Data retrieved from 28 patients (26 males, aged 3-9years (mean ± SD age: 6.1 ± 1.8years)) showed a significant improvement in IAF (9.4 Hz; p ≤ 0.025) towards 10 Hz. The CARS subdomain identified significant improvement within emotional response, object use, fear or nervousness, level of consistency of intellectual response and general impression.

The quality of life of patients significantly improved across all subclass except physical functioning. Specifically, reports from semi-structured interviews suggest improved sleep trouble – the most significant comorbidity. The experiences of minor side effects such as hyperactivity resolved within two hours following α-rTMS sessions.

In conclusion, this study presents evidence on the efficacy and safety of α-rTMS in improving ASD symptoms, quality of life and comorbid sleep troubles in children. However, these findings should be interpreted as preliminary pending the presentation of double-blind, randomised clinical trials.
